# Eruptive melanocytic nevi in a patient with amelanotic melanoma: a paraneoplastic phenomenon?

**DOI:** 10.1097/CMR.0000000000000797

**Published:** 2021-11-01

**Authors:** Vincenzo De Giorgi, Andrea Gemignani, Federica Scarfì, Luciana Trane, Flavia Silvestri, Federico Venturi, Biancamaria Zuccaro, Carmelo Urso

**Affiliations:** a Section of Dermatology, Department of Health Sciences, University of Florence, Florence; b Cancer Research ‘Attilia Pofferi’ Foundation, Pistoia; c Synlab Med - Toscana, Calenzano; d Dermatopathology Study Center of Florence, Florence, Italy

**Keywords:** dermoscopy, eruptive nevi, melanoma, paraneoplastic syndrome

## Abstract

Eruptive melanocytic nevi (EMN) describes the sudden onset of cutaneous nevi over weeks or months. Such a clinical event is generally seen in young adult patients and may be related to several possible causes. We report here a case of EMN in an old male patient followed up for a thick amelanotic cutaneous melanoma. A few months after the eruption, multiple hepatic masses, diagnosed as melanoma metastasis, were detected. The presented case may suggest that EMN may be a paraneoplastic phenomenon of alert in patients being followed for melanoma or other malignancies.

Melanocytic nevi are clonal proliferations of melanocytes, usually developing throughout childhood until the third or fourth decade of life [1,2]. In this process, in which both genetic and environmental factors are involved, a key role is generally played by the mitogenic RAS-RAF-MEK-ERK (MAPK) pathway, known to promote cellular proliferation.

Eruptive melanocytic nevi (EMN) describes the sudden onset of cutaneous nevi during weeks or months. This phenomenon is mostly seen in young adult patients and does not seem to be related to sun exposure or other triggering factors. Lesions are clinically stable over time in terms of number and color after their first appearance [1,3]. Reported causes for EMN include immunosuppression, drugs, immunosuppressive agents, bullous skin diseases, neoplastic diseases, toxin exposure, concurrent medical conditions and idiopathic factors [1,4–7].

A classification proposed by Burian *et al*., [1] introduced two categories of EMN:

(1) Widespread eruptive nevi (WEN), characterized by numerous small nevi triggered by drugs, that is, medications (ENAMs) or internal disease. The lesions are small, asymptomatic and monomorphous brown macules, most often ranging between 1 and 4 mm. The number of new nevi ranges between 10 and 2500, frequently >100. These lesions generally show no signs of clinical or dermatoscopic atypia. They have regular reticular, globular or reticular globular patterns, with a peripheral rim of brown globules representing a specific phase of melanocytic proliferation [8,9].
(2) Köbner-like eruptive nevi (KEN), in which large and sparse nevi are associated with skin diseases and most often localized at the site of a previous skin disease/trauma. These lesions may be associated with blistering diseases, such as epidermolysis bullosa, Stevens–Johnson syndrome, toxic epidermal necrolysis, erythema multiforme and bullous pemphigoid. Nevi are often reported as polymorphic, single or rare, and very large, frequently measuring a few centimeters in diameter. Satellite lesions may be seen in some cases. Fulfilling most ABCDE (Asymmetry, Border irregularity, Color variegation, Diameter >6 mm, Evolving) criteria, EMN associated with skin diseases may sometimes appear suspicious of melanoma.

We report a case of amelanotic EMN onset in a male patient with a previous thick amelanotic melanoma.

An 83-year-old man with Fitzpatrick phenotype 1 (red hair, blue eyes and untanned skin) presented with an eruption of numerous cutaneous well delimitated amelanotic lesions on his back that suddenly had appeared some weeks before (Fig. 1a). He had a personal history of repetitive sunburn and numerous basal cell carcinomas. About 3 years ago, he was diagnosed with nodular amelanotic, ulcerated, melanoma (Breslow 1.3 mm, Clark’s level IV, 4 mitoses) of the back. The axillary sentinel lymph node was negative, and no BRAF mutation was detected at the genomic analysis. The patient began a regular semestral follow-up protocol consisting of a clinical examination every 6 months for 5 years, then annual for another 5 years, ultrasound of lymphatic stations and pericicatricial area every 6 months, abdomen ultrasound every 12 months.

**Fig. 1:**
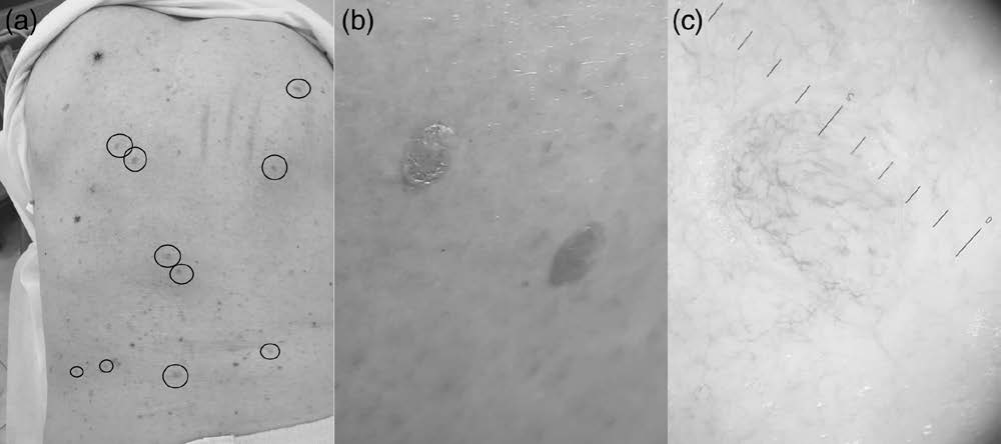
An 83-year-old man, back. Clinical and dermoscopic features. (a) Eruption of numerous cutaneous well delimitated amelanotic lesions. All the lesions appeared to have the same characteristics. (b) They were rounded, 5–6 mm in diameter, with clear and regular margins, an intensely erythematous color and slightly palpable features. (c) Dermatoscopically, they had a nonspecific pattern. No melanocytic parameters were detected; only a telangiectatic reticulum appeared to involve the entire lesion.

At the clinical level, all the lesions appeared to have the same characteristics: they were rounded, 5–6 mm in diameter, with clear and regular margins, an intensely erythematous color and slightly palpable features (Fig. 1b). Dermatoscopically, they had a nonspecific pattern. No melanocytic parameters were detected; only a telangiectatic reticulum appeared to involve the entire lesion (Fig. 1c). One of these cutaneous eruptive lesions was biopsied. Histologically, the upper dermis showed a dense collection of nevus cells (Fig. 2a) with a scarce amount of melanin pigment (Fig. 2b), embedded in collagenous fibers (Fig. 2c). Cells were uniform, round or oval, with monomorphous nuclei, showing a clear maturation. (Fig. 2c). No atypia or mitoses were detected. The histopathologic diagnosis of intradermal nevus was issued. A computed tomography (CT) scan, performed shortly after to rule out possible metastatic onset, was negative. We decided, for further precaution, to repeat the CT scan after 6 months. Three years after the diagnosis of melanoma and 6 months after the sudden appearance of nevi on the back, multiple hepatic masses and a pulmonary nodule were found on the CT scan and on echographic control. As the patient had a nonmutated BRAF gene, he is currently under nivolumab for stage IV melanoma with stabilization of the disease and does not present any complications. The amelanotic eruptive melanocytic nevi showed no reduction in number and size, but no increase in number either.

**Fig. 2:**
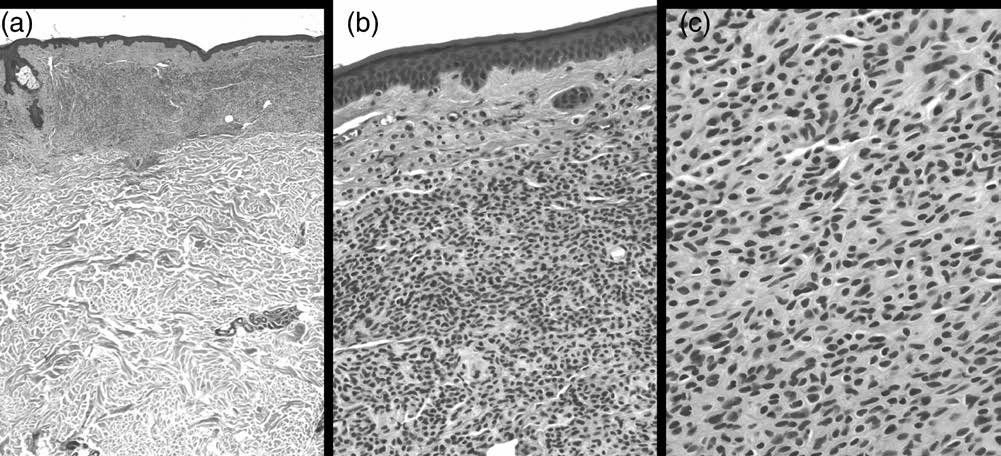
An 83-year-old man, back. Histopathologic features. (a) Nevus cells occupying the papillary dermis and extending to the upper reticular dermis (H&E ×25). (b) Uniform, round or oval cells with monomorphous nuclei and a scarce amount of melanin pigment (H&E ×200). (c) Nevus cell embedded in collagenous fibers; no evidence of atypia or mitoses (H&E ×400).

Several mechanisms have been proposed to explain the development of eruptive nevi. Most often reported causes include medications, bullous dermatoses, immunosuppression, systemic conditions and paraneoplastic disorders [7,10]. Focusing on the paraneoplastic theory, many authors have reported this phenomenon as related to skin or internal malignancies, such as concurrent primary or metastatic melanoma, prostatic cancer, interferon therapy for melanoma [7], uveal melanocytic proliferations and lung cancer [7,10–12].

Some authors have suggested that EMN may be a benign metastatic process involving the dissemination of transformed immature nevus progenitor cell; the existence of nodal nevi partially seems to support this theory. EMN has also been considered as the result of the displacement of normal melanocytes by malignant melanoma cells into the lymphatic system. This theory is known as the mechanical transport model and is supported by the fact that nevus cells may be found in the lymph nodes of melanoma patients [11].

However, the current literature and, in particular, Curth’s postulates, underlines the possible association between skin disease and an internal malignancy [13,14]. Such an association suggests the possibility that this phenomenon may be considered as a paraneoplastic syndrome. In this case, the third category of EMN may be considered, that is, eruptive paraneoplastic nevi (EPN), characterized by a sudden insurgence of pigmented or nonpigmented lesions with clinical, dermoscopic or histopathologic features of the nevi in patients with a history of malignancies. EPN should be regarded as a possible predictive factor for tumor relapse.

The following diagnostic approach may be suggested in patients with EMN.

When faced with an EMN in an adult patient, the personal oncologic history of the patient is to be evaluated: an EPN must always be considered and possibly excluded. Patients with a history of melanoma or other malignancies must undergo a re-staging of the tumor. In the absence of a personal history of cancer disease, an age-suitable cancer screening to rule out possible initial tumors (e.g. colonoscopy, mammography, PSA, PAP tests, etc.) may be performed (Fig. 3).

**Fig. 3:**
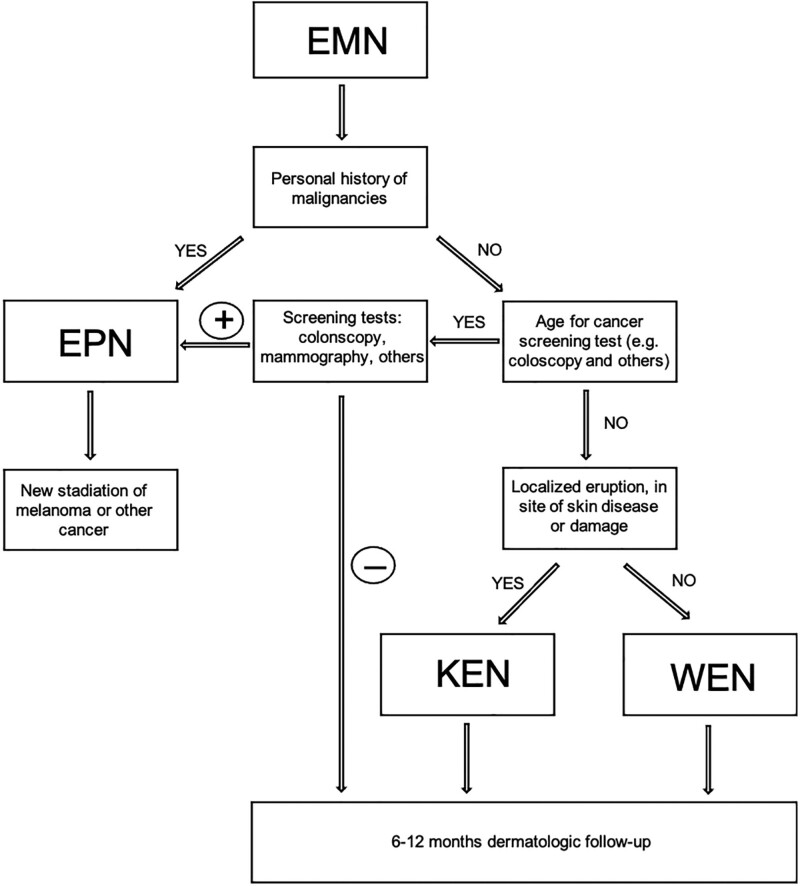
Flowchartof diagnostic behavior with a patient presenting eruptive melanocytic nevi (EMN). EPN, eruptive paraneoplastic nevi; KEN, Köbner-like eruptive nevi; WEN, widespread eruptive nevi.

To the best of our knowledge, this is the first reported case of a patient with a history of amelanotic melanoma developing amelanotic eruptive melanocytic nevi. The temporal intercurrence between the eruption and the metastasis is highly suggestive of the same pathogenetic phenomenon rather than two parallel events. This is supported by the fact that patients with pigmented melanoma have generally developed pigmented eruptions, and our patient with amelanotic melanoma but with normally pigmented nevi developed amelanotic EMN.

A topographic relation between primary melanoma and the eruption is possible, but if we consider this phenomenon a paraneoplastic event due to clones of the primary mass homing the skin, or to cytokines released from melanoma, it is admissible that this cells mediators are released in the bloodstream or in the lymphatic system, exposing all the body surface and internal viscera to them, and not only loco-regional skin.

Unfortunately, the general conditions of health of the patient, the age and the hepatic and pulmonary location of the metastasis, which were of difficult surgical approach, have made it impossible for us to perform a biopsy in the condition of safety for the patient. For this reason, BRAF testing was not performed on metastasis.

In conclusion, in patients with previous cancerous diseases, EMN should be considered as a sign of alert of a possible metastatic process. Further studies are needed to identify common mutations in the original malignancy and the eruptive nevic lesions and to establish if EMN may really be a paraneoplastic phenomenon.

## Acknowledgements

V.D.G. had full access to all data in the study and takes responsibility or data integrity and accuracy in the data analysis. Study concept and design: V.D.G and A.S. Acquisition of data: F.S., F.V., F.S., B.Z. and L.T. Analysis and interpretation of data: V.D.G. and A.S. Drafting of the manuscript: V.D.G., A.S. and C.U. Critical revision of the manuscript for important intellectual content: V.D.G. and A.S. Study supervision: V.D.G.

## Conflicts of interest

There are no conflicts of interest.
